# Supplementary material for: Systematic Genome-Wide Study and Expression Analysis of SWEET Gene Family: Sugar Transporter Family Contributes to Biotic and Abiotic Stimuli in Watermelon
Source: Int J Mol Sci. 2021 Aug 5;22(16):8407. doi: 10.3390/ijms22168407 (PMC8395094; doi:10.3390/ijms22168407)
Supplement: Supplementary file 1 [file ijms-22-08407-s001.zip › ijms-1294608-supplementary.pdf]

International Journal of *Molecular Sciences*

MDPI

Supplementary Table S1. qPCR primers used for amplification of watermelon *SWEET* genes.

Name	Gene ID	Forward primers (5'-3')	Reverse primers (5'-3')	Length
ClActin	Cla97C02G026960.1	GTCGTACAACAGGTATTGTG	AAGGTCCAGACGGAGGATAG	104
ClaSWEET1	Cla97C01G000640.1	TATGCCTCTCCATTGGTCGC	GCACGAGTTGAAGCAATCCC	190
ClaSWEET2	Cla97C01G001850.1	CGTGGCTCTCTTAGCCCTTT	CCGTTGGGGACTGTGATGAA	255
ClaSWEET3	Cla97C01G012030.1	CACCAATCCCCACATTTGCTC	GAGGACGACGGTAGCAAAGA	281
ClaSWEET4	Cla97C01G012040.1	GCCATGACCATCAAGATAGGGT	ACATGTTGTCGTCGTCCCAA	160
ClaSWEET5	Cla97C01G012050.1	TTACTCTCTCCCTCGCCAAC	TACACGTCGGACATTTGCAC	217
ClaSWEET6	Cla97C03G055120.1	TGATTCGCACAAAGAGCGTG	AGTTGTTGCATCTCCTTCGC	208
ClaSWEET7	Cla97C03G055130.1	TCTTAGCTCCTGTGCCAACATT	ATGAGACAGCCAACGGAGTT	172
ClaSWEET8	Cla97C03G067610.1	GTCTACATTGCGGTGCCAAA	ACCACCGTTCATTCCCATCT	159
ClaSWEET9	Cla97C05G087740.1	TAGGGTTTGTTTGTGCGGGA	AGTTGTGCCAATCCCAACCC	220
ClaSWEET10	Cla97C06G111200.1	ACTTAGCTCCTTTGCCGACG	GGCCCATGTTCATCACAGCA	264
ClaSWEET11	Cla97C06G127900.1	AAGGTTCCAAACGGCTAAGGT	ACGGCATGTACTCCACACTC	206
ClaSWEET12	Cla97C06G127910.1	AAGAAGGCTCGGATATTCACGG	CATAACCGCGCTGAGTGTGA	252
ClaSWEET13	Cla97C07G138130.1	TCTCCATTGCCAACCTTCGT	AGGGTTCCAGCTGCGTTAAT	170
ClaSWEET14	Cla97C08G149190.1	CCTGTCGTTGTTCGTGTTCTTG	TCGTTTGTGGGCTTCTCATCT	177
ClaSWEET15	Cla97C08G149470.1	CGTCGGCATTATTGGTAATCTCA	TCCGTCCTTTGTTATCGGCA	269
ClaSWEET16	Cla97C10G197080.1	CAATAACAAGTCGGTGGAAGAG	GGTTCCATGCAACGCTAGCA	280
ClaSWEET17	Cla97C10G197130.1	TTCTCACCCTCGGTGGAAGA	GGTTCCATGCAACGCTAGCA	279
ClaSWEET18	Cla97C10G197140.1	GCCTTTTGTTCATCCAGACAGC	CACACTTTCGTCCGTCCTCT	123
ClaSWEET19	Cla97C10G197150.1	CTTTCCCCGCTCCCAACATT	AAGCTCTATGACGAGGCCAA	183
ClaSWEET20	Cla97C11G216360.1	TTGCTCTACCAAACATAGTGGGG	TGTTGTTGTTGGTTTCTCCCA	213
ClaSWEET21	Cla97C11G216370.1	GCAGAAAAGGGTGATTACGACAA	TGCTCATCTCCTTCCTCAAGTTT	246
ClaSWEET22	Cla97C11G223200.1	AACTGACCTCCCTTTGCGAC	TGAACTATCCCCAACACGGC	246
